# Honey Bee Gut Microbiome Is Altered by In-Hive Pesticide Exposures

**DOI:** 10.3389/fmicb.2016.01255

**Published:** 2016-08-16

**Authors:** Madhavi L. Kakumanu, Alison M. Reeves, Troy D. Anderson, Richard R. Rodrigues, Mark A. Williams

**Affiliations:** ^1^Horticulture, Virginia TechBlacksburg, VA, USA; ^2^Entomology, Virginia TechBlacksburg, VA, USA; ^3^Interdisciplinary Ph.D. Program in Genetics, Bioinformatics, and Computational Biology, Virginia TechBlacksburg, VA, USA

**Keywords:** honey bee, pesticides, microbiome, miticide, chlorothalonil

## Abstract

Honey bees (*Apis mellifera*) are the primary pollinators of major horticultural crops. Over the last few decades, a substantial decline in honey bees and their colonies have been reported. While a plethora of factors could contribute to the putative decline, pathogens, and pesticides are common concerns that draw attention. In addition to potential direct effects on honey bees, indirect pesticide effects could include alteration of essential gut microbial communities and symbionts that are important to honey bee health (e.g., immune system). The primary objective of this study was to determine the microbiome associated with honey bees exposed to commonly used in-hive pesticides: coumaphos, *tau*-fluvalinate, and chlorothalonil. Treatments were replicated at three independent locations near Blacksburg Virginia, and included a no-pesticide amended control at each location. The microbiome was characterized through pyrosequencing of V2–V3 regions of the bacterial 16S rRNA gene and fungal ITS region. Pesticide exposure significantly affected the structure of bacterial but not fungal communities. The bee bacteriome, similar to other studies, was dominated by sequences derived from *Bacilli, Actinobacteria*, α-, β-, γ-*proteobacteria*. The fungal community sequences were dominated by *Ascomycetes* and *Basidiomycetes*. The Multi-response permutation procedures (MRPP) and subsequent Phylogenetic Investigation of Communities by Reconstruction of Unobserved States (PICRUSt) analysis indicated that chlorothalonil caused significant change to the structure and functional potential of the honey bee gut bacterial community relative to control. Putative genes for oxidative phosphorylation, for example, increased while sugar metabolism and peptidase potential declined in the microbiome of chlorothalonil exposed bees. The results of this field-based study suggest the potential for pesticide induced changes to the honey bee gut microbiome that warrant further investigation.

## Introduction

As pollinators and honey producers, honey bees *(Apis mellifera)* are vital to global agriculture. Over the last few decades, significant declines in bee colonies have been reported both globally and in the United States of America (USA; Cox-Foster et al., 2007; Ellis et al., 2010; vanEnglesdorp and Meixner, 2010) and major negative impacts on crop production are predicted if the trend continues (Koh et al., 2016). In addition to parasites, concerns have been raised about the effects of pathogens, poor nutrition, and pesticides on honey bees and their gut microbial symbionts (Mullin et al., 2010; Potts et al., 2010; Evans and Schwarz, 2011; Henry et al., 2012; Staveley et al., 2014; Goulson et al., 2015). High demand for honey bee services coupled with pest and disease problems have kept bee keepers reliant on miticides such as coumaphos (organophosphate) and *tau*-fluvalinate (pyrethroid) along with several in-hive medications (Elzen et al., 2000; Johnson et al., 2013) for control of infestations. There is thus a need to determine the potential influence of these commonly used chemicals on honey bees and associated gut symbionts.

Honey bees and associated gut microbiota are also exposed to pesticides applied to agricultural lands. A latest survey has identified multiple pesticide residues, both beekeeper and grower applied, in honey bee hives (Mullin et al., 2010). While in-hive application is needed to help control parasites and pathogens, the potential lethal effects of these chemicals could have unwanted side effects (Johnson et al., 2010). Exposure to pesticides has proven to be lethal even at low doses and can lead to paralysis, respiratory failure, and mortality of target (e.g., mites) and non-target (e.g., honey bees) individuals. The pesticides might also reduce the immunocompetence of honey bees, affecting overall health of the colony (Thompson, 2003; Johnson et al., 2010; Vidau et al., 2011; Frost et al., 2013; Staveley et al., 2014). Questions have been raised about the potential for changes to the gut microflora of honey bees following pesticide exposure, however field scale observations of pesticide effects at multiple locations have not been conducted.

The microbial community associated with the gut is shown to influence the growth and health of insects (Dillon and Dillon, 2004; Martinson et al., 2012). Microbial symbionts and other indigenous non-pathogenic microbial communities in the honey bee gut might be essential for sustaining the nutritional status and immunocompetence against invading pathogens (Evans and Armstrong, 2006; DeGrandi-Hoffman et al., 2010; vanEngelsdorp et al., 2010; Crotti et al., 2013). In this regard, the gut microbial community of honey bees are consistently inhabitated by bacteria belonging *to Firmicutes, Actinobacteria, and* α-, β-, *and* γ-*proteobacteria* (Jeyaprakash et al., 2003; Evans and Armstrong, 2006; Cox-Foster et al., 2007; Vásquez et al., 2009, 2012; Martinson et al., 2012; Moran et al., 2012). The consistency of occurrence of specific groups of microbes in honey bees from hives across numerous studies provide a glimpse into the potential core honey bee gut microbiota and clues to the types of microbes likely to support honey bee and hive health (Crotti et al., 2013).

A metagenomic analysis of honey bees (Engel et al., 2012) identified a number of bacterial genes that may be essential to honey bee metabolism, in particular, carbohydrate metabolism. But pesticides and in-hive medications may alter the structure and function of the microbiome and thus affect gut function and overall health of the colony (Alaux et al., 2010; vanEngelsdorp et al., 2010). In this context, little is known about the effect of in-hive pesticide residues on the honey bee microbiota and bee health. The overall hypothesis of the proposed research was that in-hive pesticide residues would alter the honey bee gut microbiome structure and function. The specific aim of this study was to investigate field-level effects that three commonly used pesticides (coumaphos, *tau*-fluvalinate, and chlorothalonil) have on the structure and potential function of the honey bee gut microbiome.

## Materials and methods

### Experimental setup

Each experimental honey bee colony consisted of a single-story hive provided with sister queens, which help to reduce genetic variation between colonies. Each hive was constructed with new frames and foundations to reduce initial exposures to disease. Three independent colonies were used for each pesticide treatment (i.e., three replicates per treatment). These hives were located at three apiaries maintained by the Department of Entomology at Virginia Tech, including Price's Fork, Kentland, and Moore Farms. Kentland is a ~3000 acre rural farm primarily used to grow fruits and vegetable crops. Price's Fork is a small (~25 acre) research farm closest to the influence of Blacksburg. Moore Farm is 250 acres, with diverse native plant populations, and surrounded by numerous farms. The experiments on each of these farms, collectively, provide a diverse and independent set of hives to determine how pesticides may affect honey bee gut microbiomes. These hives were established in May and allowed to reach colony strength by July 2012 (i.e., 6 weeks after colony establishment).

The experimental honey bee colonies included four treatments: (1) no pesticide (control); (2) *tau*-fluvalinate (Apistan®, Zoecon); (3) coumaphos (CheckMite+®, Bayer CropScience); and (4) chlorothalonil treatment. For the *tau*-fluvalinate and coumaphos treatments, honey bee colonies were treated with two *tau*-fluvalinate-impregnated or two coumaphos-impregnated strips, each containing ~10% of the active ingredient. These were utilized for 6 weeks using the manufacturer's label recommendations. The chlorothalonil treatment (10 μg/L, or parts per billion) was provided to the honey bees in a 30% sucrose solution, also for 6 weeks. In addition, the *tau*-fluvalinate and coumaphos treated and untreated honey bee colonies were also provided with a 30% sucrose solution and other identical management for the 6 week period to help isolate the effects of treatment.

Following adult emergence, random groups of honey bees were marked with Testors™ model paint to identify and collect honey bees of known age. Two random frames of brood from each colony were collected so that ~100 bees could be marked. The frames were put into custom made cages in an incubation chamber (34°C) for 6–8 h. Following emergence, bees were then exposed to pine needle smoke to eliminate paint odors. Each treatment group was marked with a distinguishable color for collection.

Following a 6 week pesticide treatment period, a random sample of brood-nest honey bees were collected from the brood frames and a random sample of foraging honey bees was collected from the hive entrance. The bee samples were collected and placed on dry ice, and then stored at −80°C until DNA extraction.

### DNA extraction

Genomic DNA was extracted from a random sample of five bees pooled for each replicate. Before DNA extraction, each bee was individually surface sterilized by rinsing in 70% and then 90% ethanol solution for 30 s each, followed by multiple washes in sterile PBS buffer and sterile water. The bees were thoroughly macerated in sterile PBS buffer using a sterile plastic pestle. The mixture was briefly centrifuged and the supernatant solution was transferred into fresh tubes. DNA was extracted using ZR soil DNA extraction kit (Cat. No: D6001, Zymo research) as per manufacturer's protocol and quantified with Nanodrop 1000 (Nanodrop, Thermo scientific). The DNA samples were stored at −20°C until further use.

Before sending the samples to sequencing, the quality of the extracted genomic DNA samples was checked by amplifying 16S gene using the 27F (AGAGTTTGATCMTGGCT CAG) and 1492R (GGTTACCTTGTTACGACTT) primers. Briefly, genomic DNA was amplified in a 25 μl reaction mix comprising 12.5 μl ImmoMix red (Catalog No. BIO-25022; Bioline), 0.5 μl MgCl_2_ (50 mM), 0.5 μl of 27F primer (10 μm), and 1492R (10 μm) primers each, 2 μl template DNA and 8 μl of nuclease free water. The protocol for the amplification was as follows: 10.0 min of initial activation at 95°C, followed by 30 cycles of denaturation at 94°C for 30 s, annealing at 48°C for 30 s, and extension at 72°C for 1 min with a final extension at 72°C for 5 min. The PCR products were electrophoresed on a 1.5% (w/v) agarose gel, stained with ethidium bromide, and bands of target DNA visualized under UV light.

### Pyrosequencing for bacteria and fungi

A total of 24 samples (comprising 2 bee type × 3 sites × 4 treatments) were sequenced individually for bacteria and fungi at the MRDNA sequencing facility (Shallow water, Texas, USA). The V2–V3 region of the 16S rRNA gene was sequenced for bacteria and the ITS region for fungi using unique barcoded primers. The 16S primers 104F (GCCTCCCTCGCGCCATCAG NNNNNNNNGGCGVACGGGTGAGTAA) and 530R (GCCTTGCCAGCCCG CTCAG CCGCNGCNGCTGGCAC) for bacteria and ITS1-F (GCCTCCCTCGCGCCATCAG NNNNNNNNCTTGGTCATTTAGAGGAAGTAA) and ITS4-R (GCCTTGCCAGCCCGCTC AGTCCTCCGCTTATTGATATGC) were used for fungi (8 bp unique barcode information provided as Data Sheet 1). Samples were amplified as per MRDNA protocols (Dowd et al., 2008) and sequenced using a Roche 454 FLX titanium instruments and reagents following manufacturer's guidelines.

### 16S rRNA gene sequence analysis

The bacterial data was analyzed in QIIME 1.8.0 (Caporaso et al., 2010). The 8 bp barcodes and reverse primers (–*z* truncate_only) were removed and reads were quality trimmed with default settings to filter out sequences with length < 200 bases, no mismatch in primer, or quality < 25. Further, analysis was performed as described previously (Rodrigues et al., 2015). The bacterial (Edgar, 2010) reads were binned into operational taxonomic units (OTUs) using an open OTU-picking strategy with 97% similarity and taxonomic assignment using uclust against the Greengenes reference database v13.8 (DeSantis et al., 2006; McDonald et al., 2012). Downstream analysis was performed using a sampling depth of 1000 sequences/rep/treatment. Beta diversity of the bacterial communities were calculated using weighted Unifrac (Lozupone and Knight, 2005), used for Principal Coordinate Analysis, and further used to identify whether treatments significantly affected the microbial sequence abundance and composition using PERMANOVA (vegan v2.0.10) Using Distance Matrices (Anderson, 2001) and Analysis of Similarity (ANOSIM; Clarke, 1993). Chao1 index (Hill et al., 2003) and number of observed species were used to describe richness. Relative abundances of bacterial orders from bees exposed to different pesticide treatments were compared using a Mann Whitney *U*-test followed by Benjamini-Hochberg correction (*q* < 0.05).

Functional inference of the bacterial community was made by PICRUSt analysis (Langille et al., 2013) of the OTUs obtained from the Greengenes reference database. OTUs not part of the closed reference OTU picking were filtered out and the actual abundance of remaining OTUs utilized the default parameters for PICRUSt analyses. Using STAMP (Parks et al., 2014), two-sided Welch's *t*-test (Welch, 1947) with Benjamini-Hochberg (Benjamini and Hochberg, 1995) multiple testing correction were performed to identify Level-3 KEGG pathways that were significantly different (*q* < 0.05) between groups.

### ITS sequence analysis

The ITS sequence data was analyzed in QIIME 1.8.0 (Caporaso et al., 2010). The 8 bp barcodes and reverse primers (–*z* truncate_only) were removed and reads were quality trimmed with default settings to filter out sequences with length < 200 bases, no mismatch in primer, or quality < 20. Further analysis was performed as described previously (Rodrigues et al., 2015). The fungal reads were binned into OTUs using an open OTU-picking strategy with 97% similarity and taxonomic assignment using RDP classifier (Wang et al., 2007) against the UNITE (Abarenkov et al., 2010) reference database v12.11. Downstream analysis on the ITS data was performed at sequence depth of 295 sequences/rep/treatment as described above in 16S rRNA gene sequence analysis; whereas the beta diversity of the fungal communities were calculated using Bray Curtis (Beals, 1984) distance and used for Principal Coordinate Analysis.

The sequence data files were deposited in NCBI SRA database under bioproject PRJNA320132 with biosamples SAMN04917371 (16S) and SAMN04917372 (ITS) and accession numbers SRR3467967 and SRR3467969.

## Results

A total of 144,638 16S rRNA gene and 96,373 ITS sequences were obtained from pyrosequencing 24 samples (4 pesticide treatments × 3 sites × 2 bees types), each sample comprising pooled DNA from five honey bees. For 16S data, 47,965 quality reads with a minimum length of 200 bp were retained after stringent quality filtering. The number of sequences averaged 1989 (±847). OTU picking of the 16S data at the 97% similarity gave 340 OTUs across all samples. A threshold of 1000 sequences of subsampling resulted in removing a sample with low number of reads (Foragers treated with chlorothalonil at Price's Forks site, 205 sequences) from the downstream analysis (Tables S1, S2, S3).

Similarly, for fungi, 24,214 reads were retained from 97,373 ITS sequences after stringent quality checking. All the quality reads were clustered into 555 OTUs (97% similarity). Rarefaction for both the 16S and ITS data (Image 1) showed a plateau supporting the estimates of richness.

### Bacterial and fungal diversity and composition in response to pesticides

**Figure 1:**
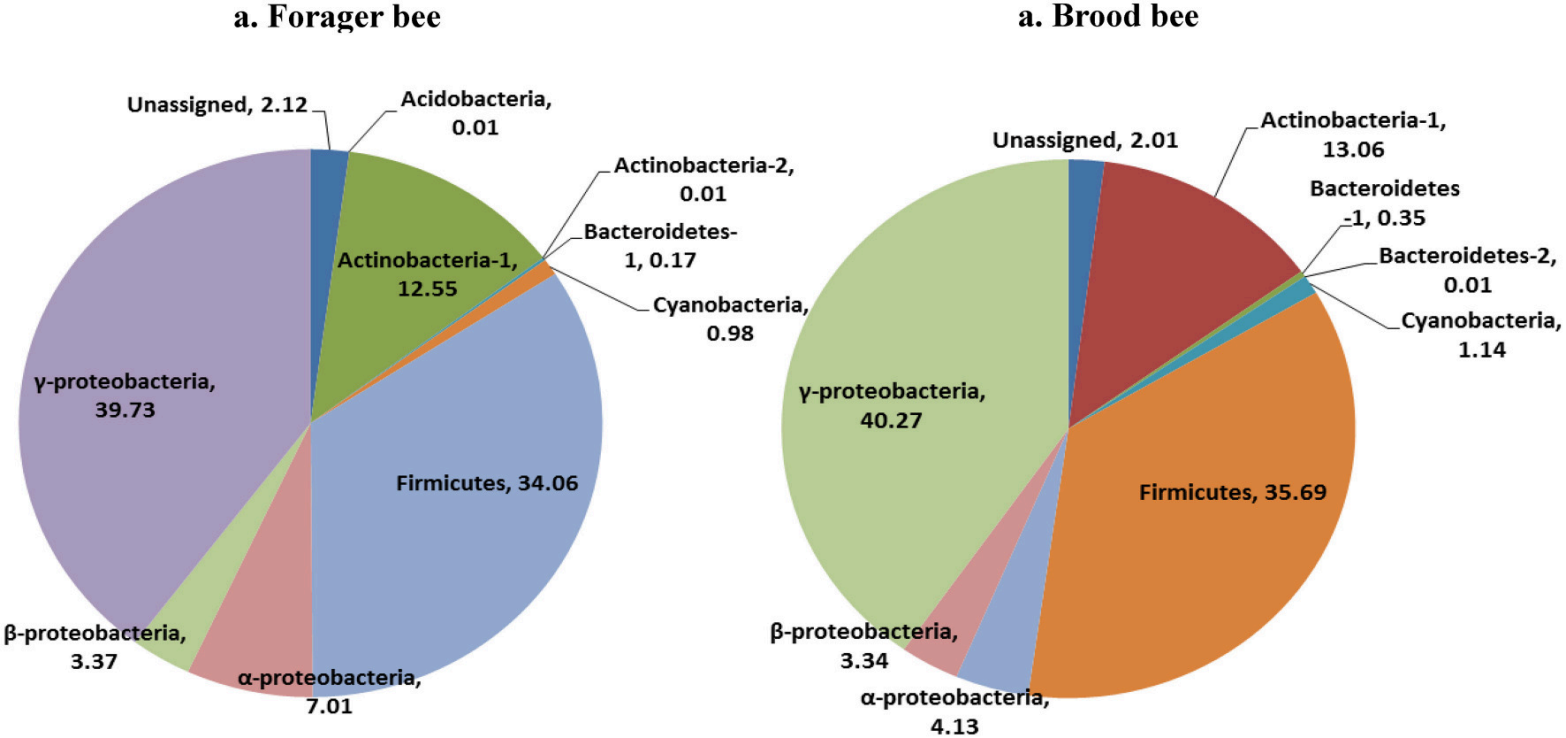
**Relative abundance of bacterial taxa (class) in forager bee (***n*** = 11) and brood bees (***n*** = 12)**.

**Figure 2:**
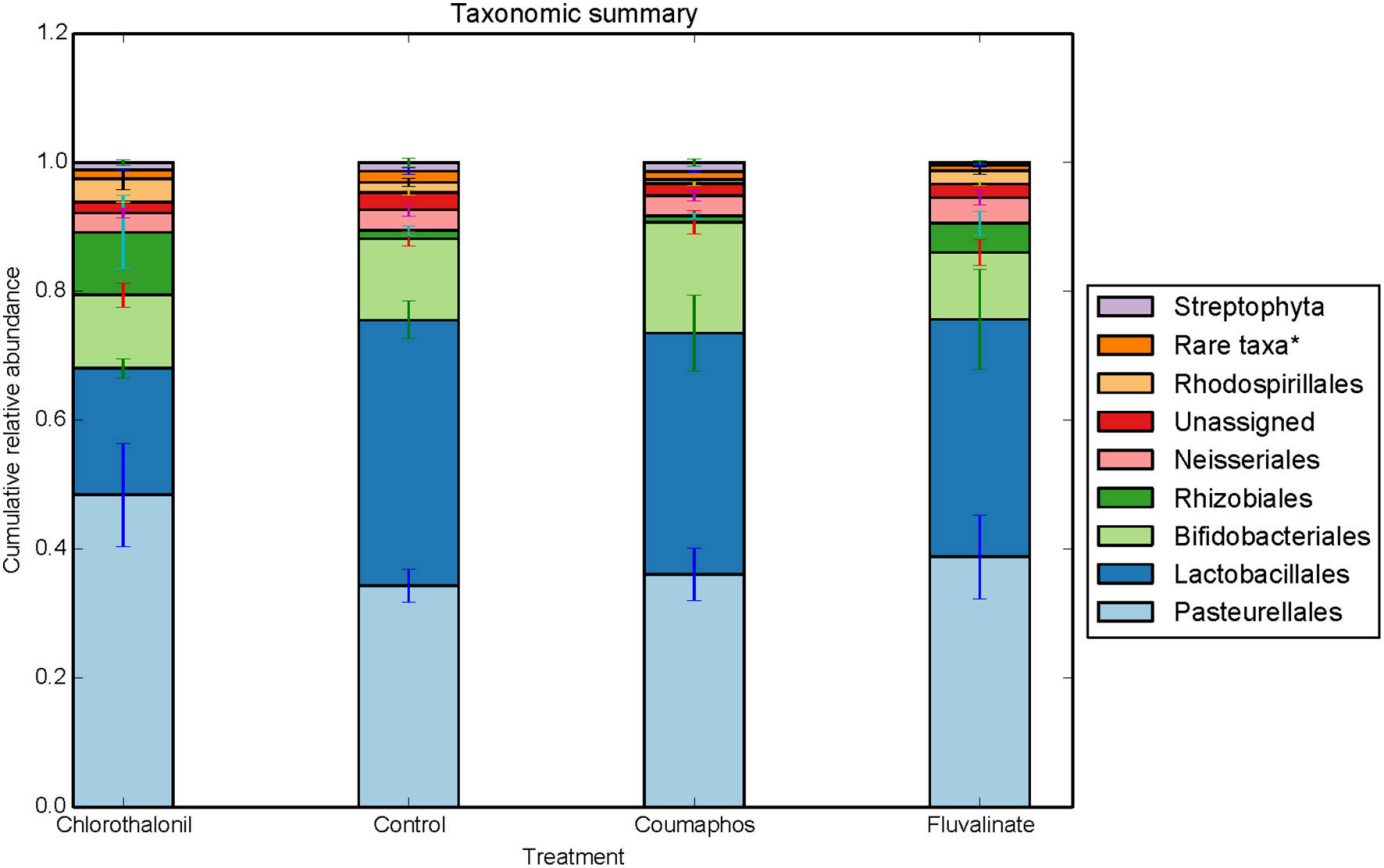
**Relative abundance (cumulative with error bars) of bacterial taxa (order) in bees exposed to different pesticide treatments in three experimental sites**. Each column in the graph is the average (*n* = 6) of the percentage abundance of each taxa in each pesticide treatment. “Rare taxa^*^ indicates orders that were < 1% in average abundance across all samples.”

Greengenes database used for the taxonomic assignment of 16S data is described below. Overall, the bacterial diversity and composition in forager and brood bees was consistent irrespective of the treatment (Figure 1), and data therefore were combined to describe the influence of pesticide treatment on community structure. As per the taxonomic assignment using Greengenes the honey bee gut bacteriome was dominated by sequences from *Proteobacteria* (49.2%), *Firmicutes* (34.4%) and *Actinobacteria* (13.0%), comprising 97% of the data (Table S1). The bacterial community was dominated by members of family *Lactobacillaceae* (34%) of class *Bacilli* (34.4%), *Bifidobacteriaceae* (12.5%) of class *Actinobacteria* (12.8%), and members of class γ-*proteobacteria* (39.7%) (Figure 2). The sequences from γ-*proteobacteria* were predominantly dominated by member of order *Pasteurellales*. Along with *Pasteurellales*, reads assigned to *Enterobacteriales* (genera *Enterobacter, Serratia*, and *Morganella*) and *Pseudomonadales* were also commonly observed. Likewise, members of α-*proteobacteria* (5.9%) and β-*proteobacteria* (3.6%) were predominantly assigned to the family *Neisseriaceae* and *Bartonellaceae*, respectively.

**Figure 3:**
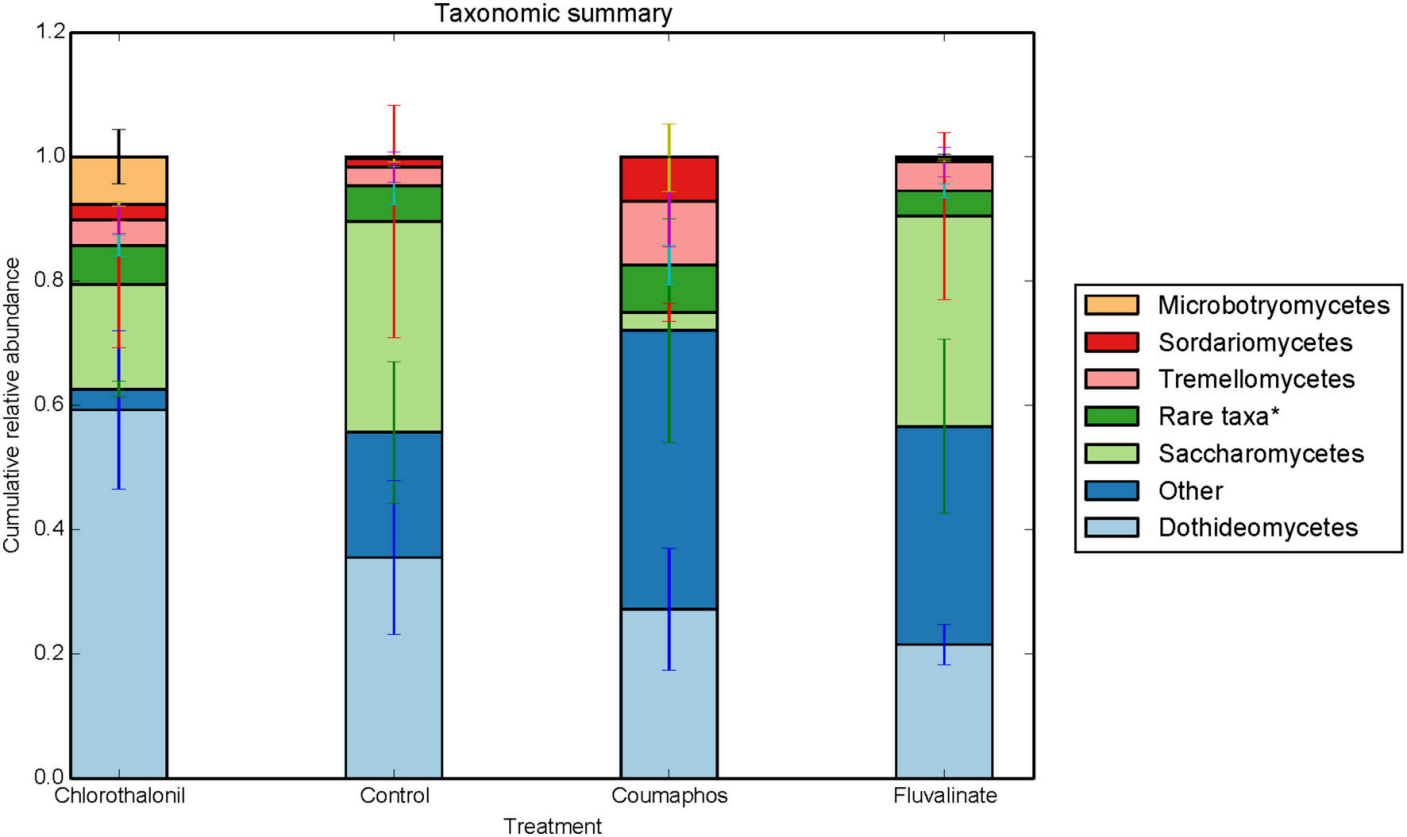
**Relative abundance of fungal taxa (class) in control, coumophos, fluvalinate, and chlorothalonil treated honey bees**. Each column in the graph is the average (*n* = 6) of the percentage abundance of each taxa in each pesticide treatment. “Rare taxa^*^ indicates classes that were < 2% in average abundance across all samples.”

Overall, the fungal OTUs predominantly belonged to phyla *Ascomycota* (64.6%) and *Basidiomycota* (9.4%). The fungal members of unspecified class (25.8%) were also identified but confined to few treatments (Table S1). It is to be noted that there is large variation in the community composition among different treatments. For instance, the composition of *Ascomycota* ranged from 34 to 85% of taxa depending upon treatment. The phylum *Ascomycota* were dominated by fungal sequences belonging to classes *Dothideomycetes, Saccharomycetes*. Class *Tremellomycetes* was the predominant member of *Basidiomycota*, comprising nearly 63% of the total (Figure 3). The sequences were most commonly associated with fungal genera such as *Metschnikowia, Alternaria, Cladosporium*, some unspecified members of *Mycosphaerellaceae* and *Cryptococcus*.

### Microbial community structure changes associated with pesticide exposure

**Figure 4:**
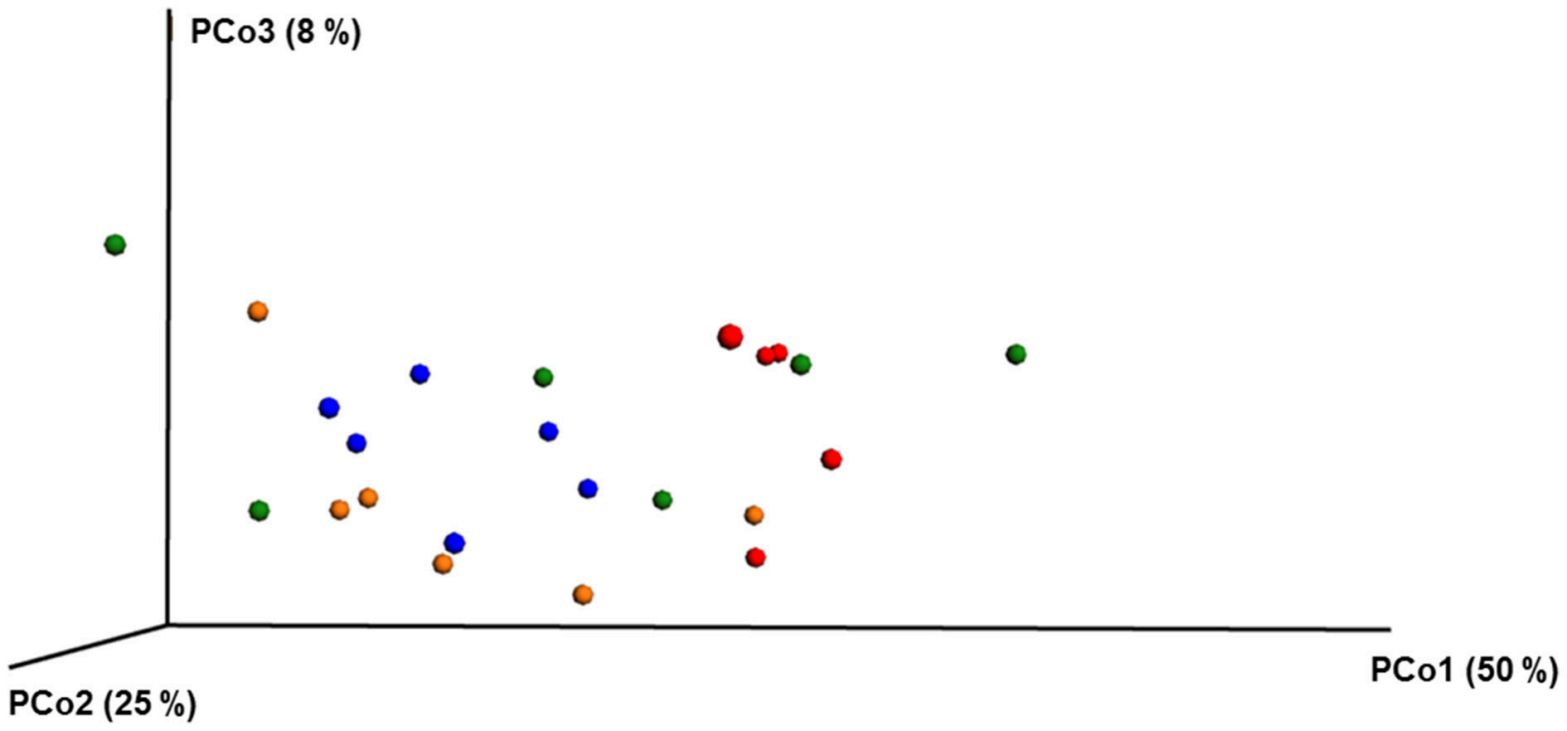
**PCoA plot with weighted unifrac metric showing the distribution of bacterial community composition in honey bees exposed to different pesticide treatments across three different locations**. Each circle with same color indicate the bees receiving the same pesticide treatment [Chlorthalonil (Red), Control (Blue), Coumaphos (orange), and *tau*-fluvalinate (green)] from three locations. Adonis and anosim were used to analyze the significant differences between the treatments.

ANOSIM and MRPP on weighted Unifrac distance, of the bacterial OTUs showed the significant effect of pesticides (*P* < 0.05) on the honey bee bacteriome, however, chlorothalonil, in particular, had the largest impact on bacterial community structure relative to the other pesticide treatments (*P* < 0.05). Location of the bee hives alone had not significantly affected the bacteriome but the interaction of site x pesticide treatment has significant impact on the bacterial structure (*P* < 0.01) but not diversity (Table S4). At the order level (Figure 2), significant differences (Benjamini-Hochberg, *q* < 0.05) were observed in the relative abundances of certain bacterial groups within the pesticide treatments and relative to control treatment. The abundance of *Lactobacillales* in chlorothalonil treated bees was significantly lower as compared to control and coumaphos treatments, whereas *Burkholderiales* decreased in control and increased in coumaphos treatments, respectively (Figure 2). The relative abundance of *Bifidobacteriales* was higher in Coumophos compared to others. Also, *Rhodocyclales* significantly varied in the coumaphos treatment as compared to *tau*-fluvalinate or control and similarly, the abundance of *Enterobacteriales* and *Caulobacterales* were different in *tau*-fluvalinate as compared to chlorothalonil or control. Overall, there were differences in bacterial community associated with chlorothalonil treated bee colonies relative to control (Figure 4).

**Figure 5:**
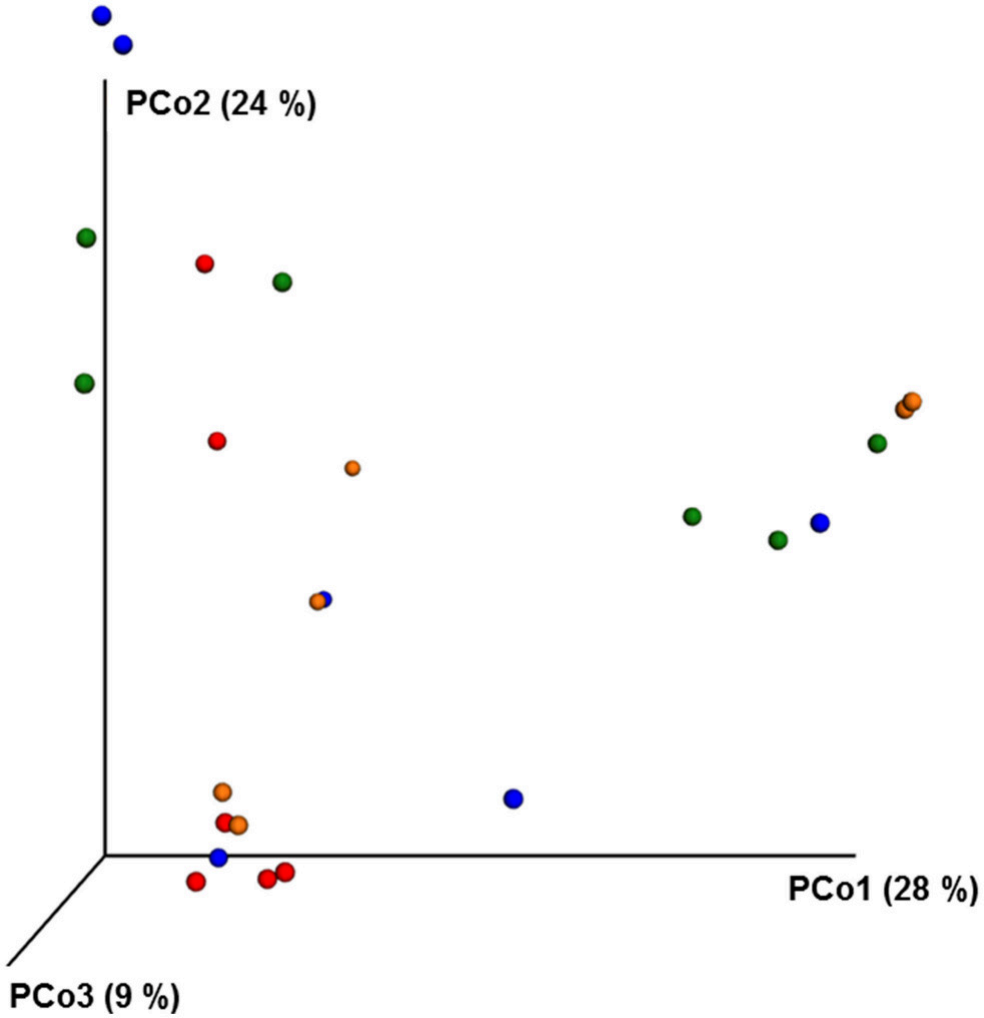
**PCoA plot with Bray-Curtis distance showing the distribution of fungal community composition in honey bees exposed to different pesticide treatments across three different locations**. Each circle with same color indicate the bees receiving the same pesticide treatment [Chlorthalonil (Red), Control (Blue), Coumaphos (orange), and *tau*-fluvalinate (green)] from three locations. Adonis and anosim were used to analyze the significant differences between the treatments.

ANOSIM and MRPP analysis of the ITS data using weighted Unifrac distance, showed the significant effect of site, and the interaction of site × pesticide treatment (*P* < 0.05) on the honey bee fungal community, but the pesticide treatments alone did not have any significant impact on the fungal community structure. The PCoA of the ITS data (Figure 5) and diversity (Table S5) reflected this trend where samples primarily clustered by location. The effect of pesticide treatment on fungal communities was less clear (Figure 5). Overall, there was little evidence that fungal communities of honey bees were changed due to pesticide exposure.

### Functional inferences of the bacteriomes

**Figure 6:**
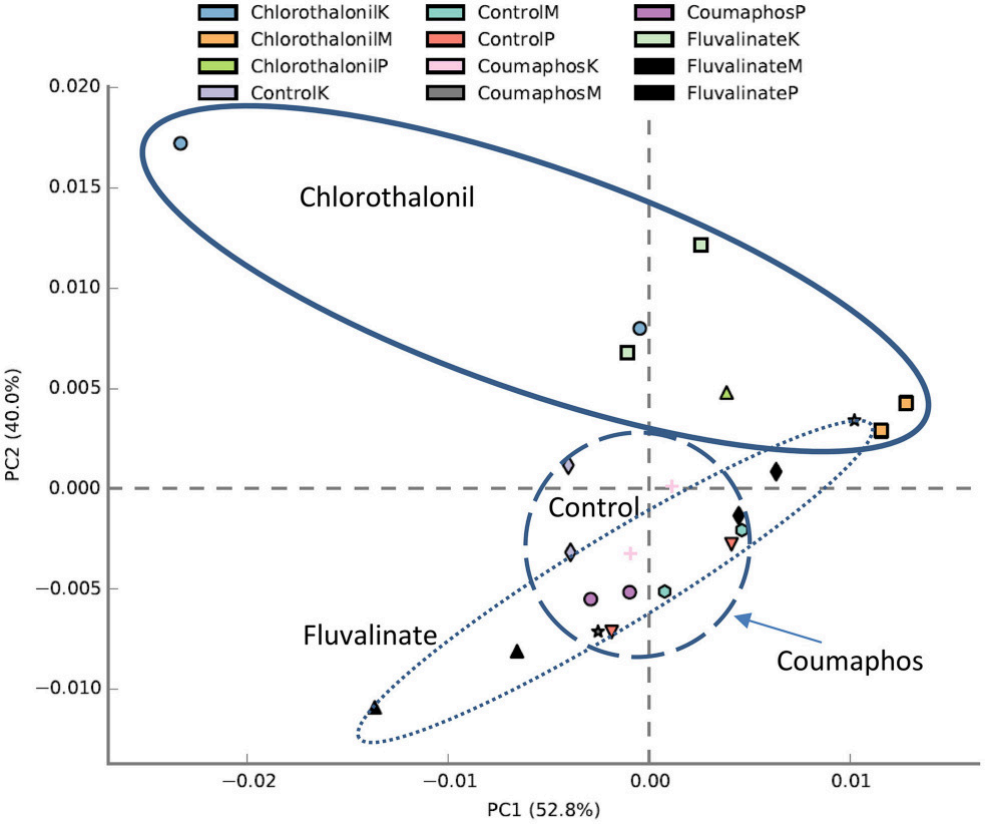
**PCA plot describing functional inferences (PICRUSt) of bacterial communities across pesticide treatments**. Each circle with same color indicates the forager and brood receiving same pesticide treatment in a given site. Ovals help to outline the extent of each treatment. K, M, and P in the legend represents Kentland Farm (K), Moore (M), and Price's Fork (P) locations respectively where the experiment was conducted.

**Figure 7:**
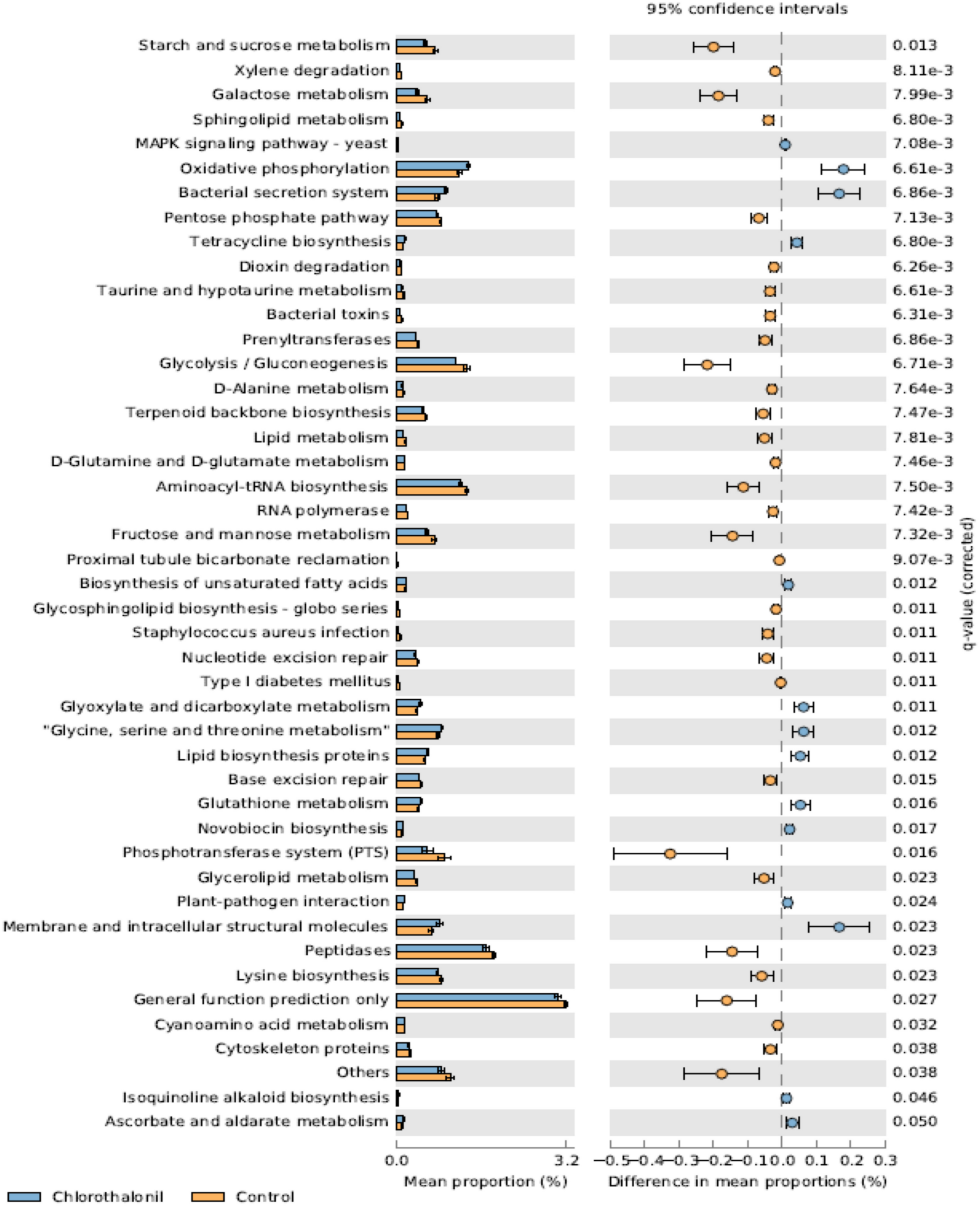
**Mean proportion (%) and the difference in the mean proportion (%) of predicted and significantly different (Welch's ***t***-test, Benjamini-Hochberg; ***q*** < 0.05) functional inferences of honey bee microbiota in chlorothalonil (blue) and control (orange) treatments**.

PICRUSt analysis predicted 6909 enzymes in the normalized data of bacterial sequences. These enzymes are categorized into 328 KEGG pathways associated with key metabolic functions related to energy, carbohydrate, amino acid, and lipid metabolism. The functional profile was observed to change with pesticide treatments (Figure 6) however; the statistical significant effects were related to chlorothalonil amendment (Figure 7).

## Discussion

This study aimed to determine the microbiome structure and function of honey bees exposed to in-hive pesticide residues, coumaphos, *tau*-fluvalinate, and chlorothalonil. There was a significant effect of pesticides on bee associated bacterial community structure. The alterations in bacterial community structure might be linked to changes in bacterial function. The functional inferences of PICRUSt showed increased oxidative phosphorylation while KEGG functions related to sugar metabolism and protease activity decreased as a result of the pesticide chlorothalonil. The results highlight the potential that changing microbial community structure may have on the functional ability of the microbiome to metabolize sugars and peptides, presumably vital processes contributing to honey bee health. These results thus point to the need to directly measure functional metabolic changes (e.g., sugar and peptide metabolism) within the honey bee gut in response to pesticide exposure and the potential ramifications of this change to honey bee health.

Overall, *Firmicutes, Actinobacteria*, and *Proteobacteria* were the dominant bacterial phyla of the honey bees. In particular, the bacteria belonging to *Lactobacillaceae, Bifidobacteriaceae, and* γ-*proteobacteria* were abundant and the results were consistent with previously reported culture-dependent and independent studies (Hamdi et al., 2011; Martinson et al., 2012; Crotti et al., 2013). There were however, changes in the relative abundance of several taxa that help to explain the effects of pesticides [e.g., chlorothalonil (Figure 2)]. Vulnerability of immature honey bees to chlorothalonil in this regard was recently reported (Zhu et al., 2014). The relative abundance of *Lactobacillaceae*, in our data, was observed to decline in chlorothalonil-treated colonies compared to un-treated colonies. The *Enterobacteriaceae* and *Caulobacteraceae*, in contrast, exhibited a relative increase in association with chlorothalonil compared to *tau*-fluvalinate. The changes in bacterial community structure may alter the gene expression and community function and thus could have ramifications for honey bee activities, physiology, and behavior (Engel and Moran, 2013). Bacteria belonging to classes *Bacilli*, γ-*proteobacteria, and Actinobacteria*, for example, were predicted to participate in the breakdown and fermentation of macromolecules such as polysaccharides and polypeptides (Lee et al., 2015). The bacterial ability to catabolize these molecules and generate various fermentation products, such as short-chain fatty acids and alcohols (Lee et al., 2015), could be affected by change to bacterial communities of the honey bee gut.

Members of class *Bacilli* and *Actinobacteria*, in particular, genera *Lactobacillus* and *Bifidobacterium* are known beneficial gut symbionts in many organisms, including honey bees and humans. In honey bees, these bacteria are thought to be involved in nectar processing (Vásquez et al., 2012), carbohydrate metabolism, immunomodulation, and pathogen interference. The dynamics in structure and, in some cases, the relative reduction in specific gut microbiota were consistent with the effect that pesticides have been hypothesized to have on beneficial gut bacteria in the honey bees (Anderson et al., 2011).

Members of *Orbaceae* and *Neisseriaceae* such as *Gilliamella apicola, Frischella*, and *Snodgrassella alvi* were reported in honey bee guts in several recent studies (Martinson et al., 2012; Moran et al., 2012; Horton et al., 2015; Tarpy et al., 2015). These bacteria were described as part of core microbiome of the honey bee gut; however, the functional roles of these bacterial groups are not yet fully understood. The gram negative bacteria belonging to class γ- and β-*proteobacteria* belonging to *Pasteurellales* and *Neisseriaceae* were observed in our study. Even though no taxa were assigned to the class *Orabales* in our analysis, *Pasteurallaes* are considered phylogenetically very similar to *Orabales* (Kwong and Moran, 2013). A deeper understanding of the rRNA and genomic sequences of these organisms will help to clarify there phylogenetic differences, and relevance to gut function.

Other shifts in the honey bee gut microbiota could also influence the nutrition, immunity, and overall health of the bees. Many members of class *Enterobacteriaceae*, for example, are facultative anaerobes involved in sugar fermentation and nitrogen metabolism (Anderson et al., 2011) and so changes in the relative abundance of these taxa could disrupt honey bee metabolism. Other groups of bacteria belonging to the genera *Serratia, Edwersiella, Acetobacter, Mannheimia, Gluconobacter, Bartonella*, and *Klebsiella* (Jeyaprakash et al., 2003; Engel et al., 2012) were observed, however, the importance of these bacteria to honey bee health is not well-known. *Bartonella*, in this regard, is a known opportunistic pathogen, and its presence may indicate an antagonistic role. Results of the current study suggest that pesticides such as chlorothalonil have the potential to alter the gut microbiota and its function.

Similar to bacteria, fungi can have both mutualistic and antagonistic functional roles. The bees were predominantly associated with members of *Ascomycota*, followed by *Basidiomycetes* and members of unspecified class. Although no significant effects were observed, the high within treatment variation makes it difficult to come to firm conclusions about the effects of pesticides on fungal community structure. Further, studies that attempt to elucidate pesticide effects on microbial communities of the honey bee gut, should if possible; also include descriptions of fungal communities.

Fungal taxa might play potentially important roles in the honey bee gut. *Saccharomycetes* were observed to be associated with honey bees, and they have been described as fermentors in the gut, bee bread, and other honey bee habitats (Crotti et al., 2013). A few members of the family *Dothideomycetes* are known to be pathogenic fungi (Ohm et al., 2012), but the broader role that this family may play in honey bee function and physiology is not fully known. Other fungi, related to *Metschnikowia*, a relatively slow growing fermentor known for its production of acid proteases, were observed in the community surveys, and serve as an example of a fungal group that if impacted by pesticide exposure, could have important ramifications for digestive process of honey bees.

In addition to considerations that fungi (and bacteria) play in honey bee physiology and metabolism, there may also be important affects that pesticides might have on microbes as mediators of ecosystem structure and function through mutualistic and antagonistic effects. (Gemma and Koske, 1988). Pesticides, for example, could be hypothesized to disrupt honey bee dispersal of microbes throughout ecosystem habitats. In turn, there could also be affects that disrupt the accrual of environmental microbes by honeybees. It thus should be considered that relationships between microbes and eukaryotic hosts such as honey bees may play broader roles related to ecosystem services. Overall, the variations in the relative abundance of these fungi showed weak but possibly important patterns of change related to pesticide treatment. Further, research is needed to determine the impacts that pesticides have on honey bee fungal microbiomes and how they could feedback to alter honey bee health.

Chlorothalonil was previously shown to be a commonly observed fungicide in pollen and beehives (Zhu et al., 2014). Honey bees, moreover, that were fed pollen containing chlorothalonil were three times more susceptible to *Nosema* infection (Pettis et al., 2012; Wu et al., 2012). We did not observe obvious pathogens, such as *Nosema* or *Ascophora apis*, the casual organism of chalk board disease in the honey bee microbiome. However, the methods used in this study are not likely to be ideal for observing these microbial taxa.

## Conclusion

Overall, chlorothalanil-treated hives had structurally different bacterial community compared to non-exposed colonies. Similarly, there were shifts in the community functional potential that was most evident in the chlorothalonil-treated honey bee hives. The results of this field-based study suggest the potential for pesticide induced changes to the honey bee gut microbiome, and thus warrant further investigation into whether chlorothalonil or other pesticide exposure can have biologically significant impacts on honey bee function, health, and survival.

## Author contributions

TA and MW designed the research. MK and AR performed the experiments. MK, AR, RR, and MW performed data analyses. All authors contributed in the data interpretation and manuscript writing. All authors read, commented, and approved the final version of the manuscript.

### Conflict of interest statement

The authors declare that the research was conducted in the absence of any commercial or financial relationships that could be construed as a potential conflict of interest.

## Supplementary material

Data Sheet 1 **An excel file with treatment details and 8 bp barcode information used to tag ITS and 16rRNA gene**.

Table S1 **The composition of 16S rRNA gene and ITS sequences at phylum level (relative abundance) across treatments (Excel file)**.

Table S2 **Summary of 16S rRNA gene biom table across different treatments (Excel file)**.

Table S3 **Summary of ITS region biom table across different treatments (Excel file)**.

Table S4 **The alpha diversity indices of bacterial OTUs across different treatments**.

Table S5 **The alpha diversity indices of fungal OTUs across different treatments**.

Image 1 **Rarefaction plots of bacterial and fungal alpha diversity of samples receiving different pesticide treatment using (A) chao1 and (B) observed species**.
