# Supplementary figures and images for: Honey Bee Gut Microbiome Is Altered by In-Hive Pesticide Exposures
Source: Front Microbiol. 2016 Aug 16;7:1255. doi: 10.3389/fmicb.2016.01255 (PMC4985556; doi:10.3389/fmicb.2016.01255)

(a) 16S

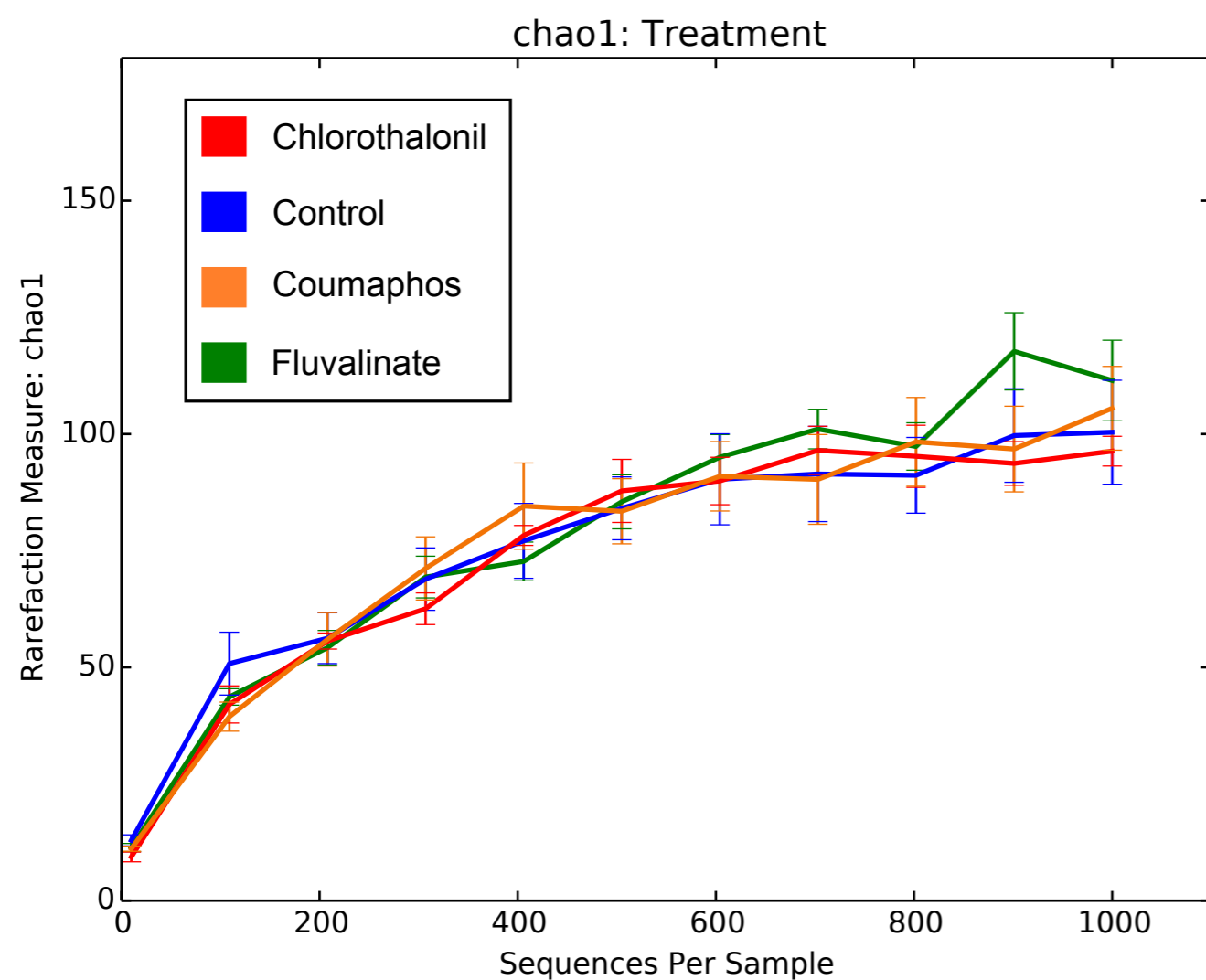

(b) ITS

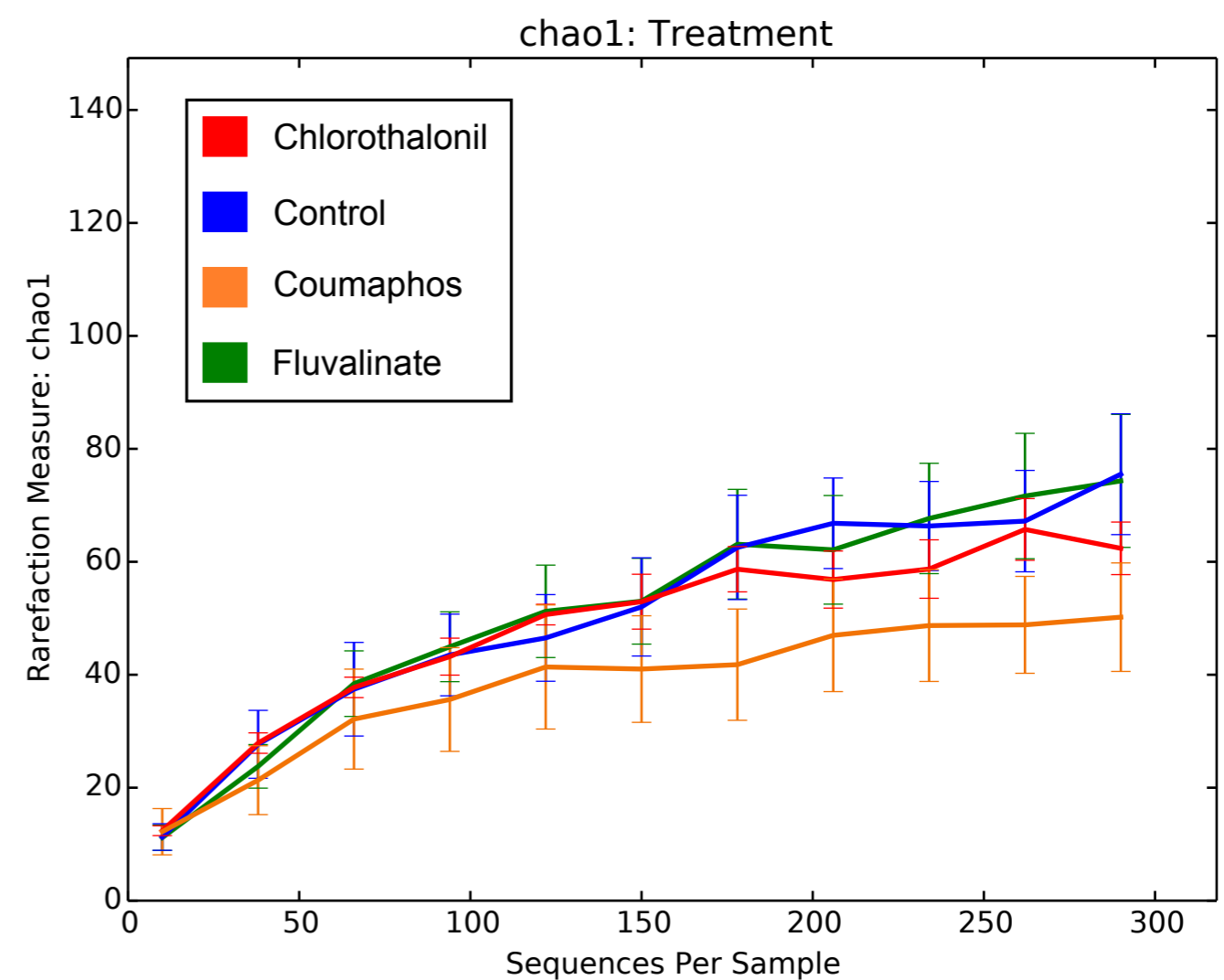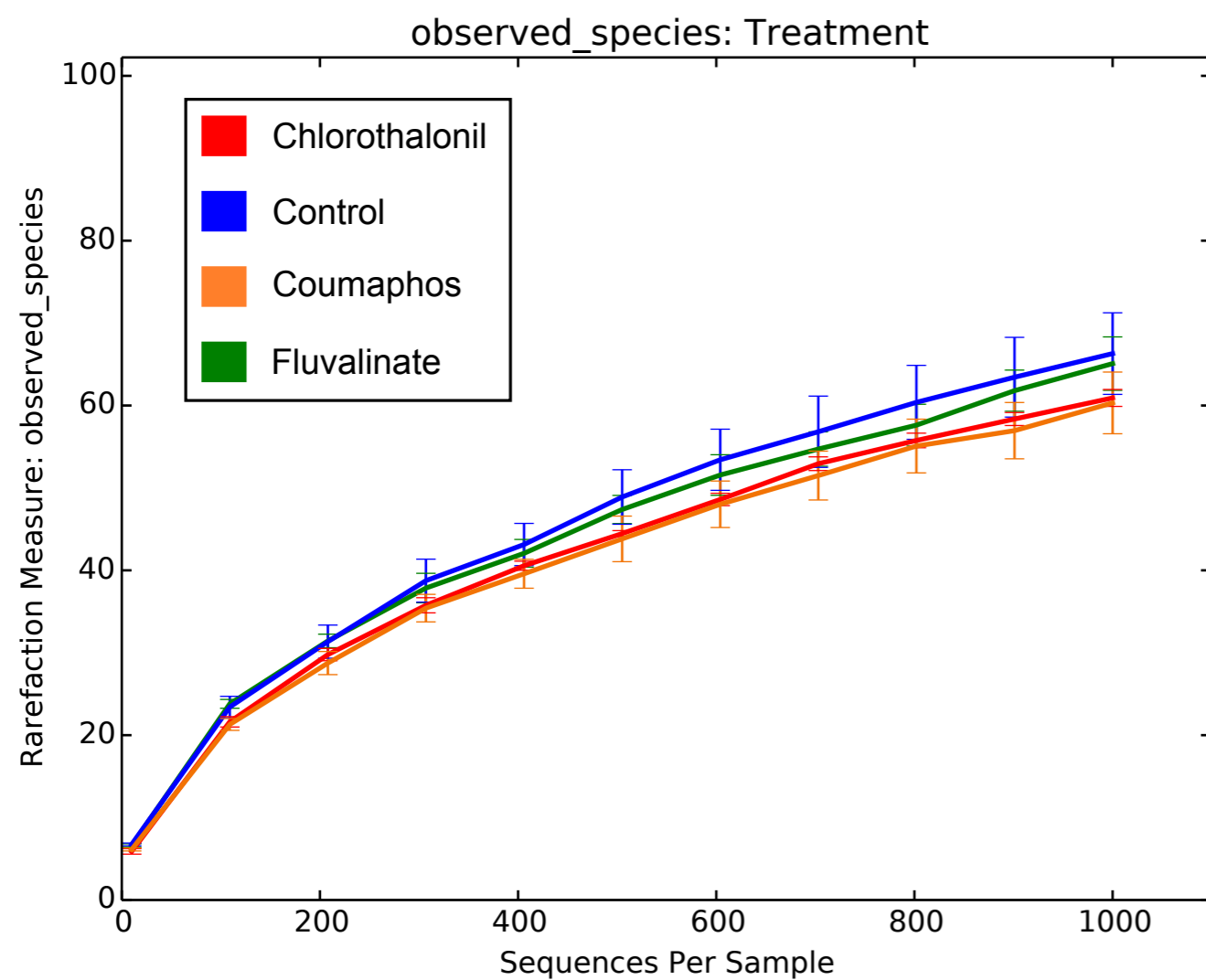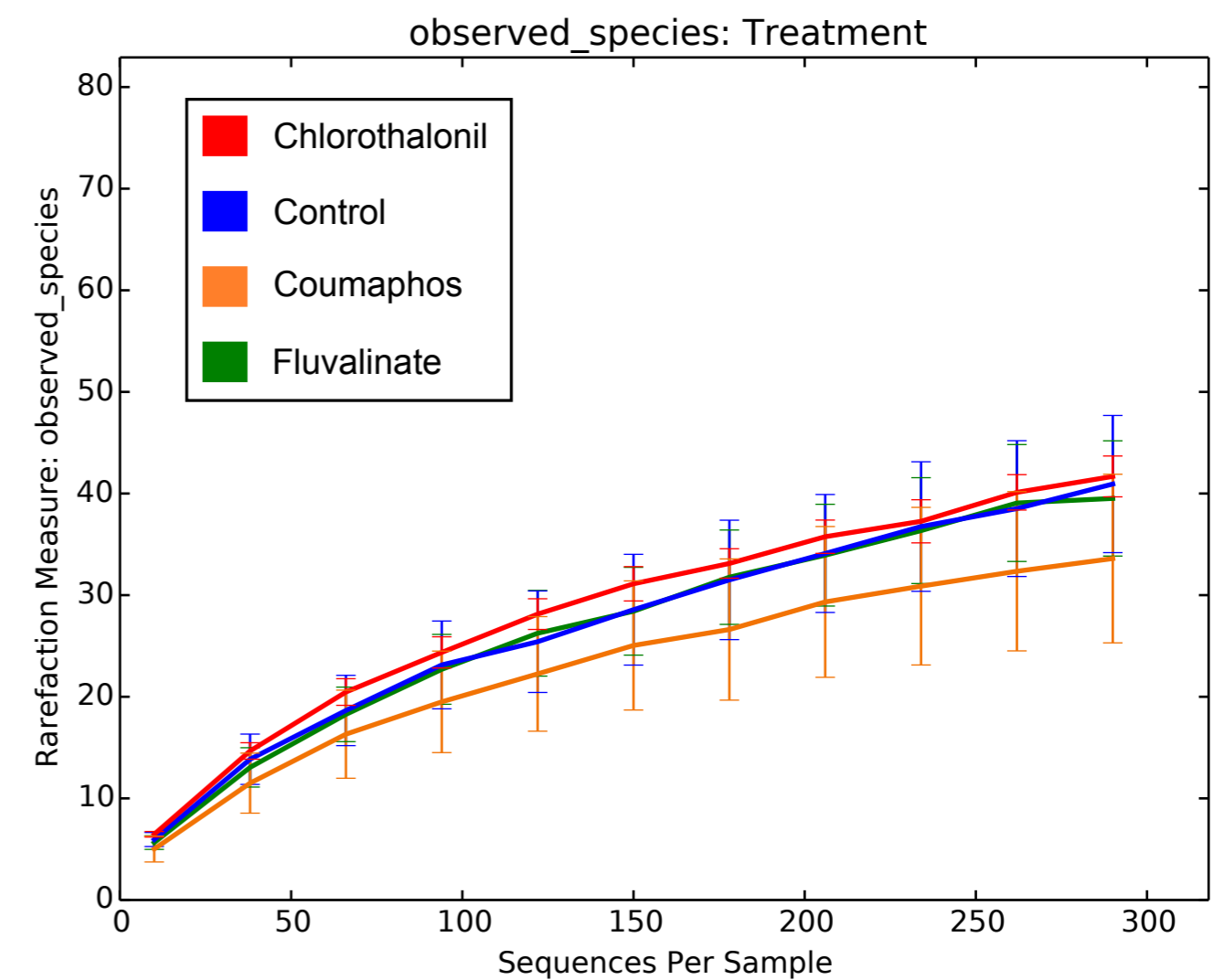

Supplement: Image 1 — Rarefaction plots of bacterial and fungal alpha diversity of samples receiving different pesticide treatment using (A) chao1 and (B) observed species. [file Image1.PDF]
